# Outcome of buccal mucosa graft urethroplasty: a detailed analysis of success, morbidity and quality of life in a contemporary patient cohort at a referral center

**DOI:** 10.1186/s12894-019-0449-5

**Published:** 2019-03-18

**Authors:** Armin Soave, Luis Kluth, Roland Dahlem, Amelie Rohwer, Michael Rink, Philipp Reiss, Margit Fisch, Oliver Engel

**Affiliations:** Department of Urology, University Medical Center Hamburg-Eppendorf, Martinistraße 52, 20246 Hamburg, Germany

**Keywords:** Urethra, Urethral stricture, Buccal mucosa, Reconstructive surgical procedure, Treatment outcome

## Abstract

**Background:**

To evaluate outcome of buccal mucosa graft urethroplasty (BMGU) for the treatment of urethral stricture disease, including a detailed analysis of success, morbidity and quality of life (QoL).

**Methods:**

Between 12/05/2008 and 07/21/2010, 187 patients with urethral stricture disease, who were treated with BMGU at our University Medical Center, received a standardized questionnaire, evaluating postoperative success, morbidity and QoL. The primary endpoint was the success, i.e., stricture recurrence-free survival plus patients’ satisfaction with surgery. Secondary endpoints included erectile function, voiding symptoms, pain and health-related QoL, which were assessed with a modified Urethral Stricture Surgery Patient Reported Outcome Measure (USS PROM), including the Erectile Function domain of the International Index of Erectile Function (IIEF-EF), Incontinence Questionnaire Male Lower Urinary Tract Symptoms Module (ICIQ-MLUTS) and EuroQol-5 dimensions (EQ-5D).

**Results:**

In total, 83 patients (51.9%) completed the questionnaire. Bulbar, penile and panurethral strictures were found in 69 patients (83.1%), 13 patients (15.7%) and one patient (1.2%), respectively. The median length of the stricture was 5 cm (range: 1–16). At a median follow-up of 46 months (range: 36–54), 65 patients (78.3%) had no stricture recurrence and were satisfied with BMGU. Median scores for ICIQ-MLUTS, IIEF-EF and EQ-5D visual analogue scale were 6, 22 and 80, respectively. Based on USS PROM, postoperative improvement of QoL and satisfaction with BMGU was found in 67 patients (80.7%) and 68 patients (81.9%), respectively.

**Conclusions:**

In patients with urethral stricture disease, BMGU offers excellent success, morbidity and QoL.

## Background

Substitution urethroplasty is the gold standard treatment for long primary urethral strictures and recurrent urethral stricture disease [1]. Depending on the stricture’s length, location and etiology, various single-stage and two-stage techniques for substitution urethroplasty have been successfully established in daily clinical practice [2], including ventral onlay [3], dorsal inlay [4] or modified procedures [5]. Due to its favorable availability, simple processing and durable integration in the urethra [6], autologous buccal mucosa currently remains the most commonly used transplant for substitution urethroplasty [1, 7].

Several studies confirmed excellent stricture recurrence-free survival of buccal mucosa graft urethroplasty (BMGU) for the treatment of USD [5, 8]. In contrast, outcome comprising morbidity and health-related quality of life (QoL) has been poorly investigated thus far. In addition, previous studies included heterogeneous patient cohorts, various urethroplasty procedures and mainly lack of standardized validated instruments such as patient-reported outcome measures (PROM) [9–11], which might complicate the comparability of results. In 2011, Jackson et al. introduced Urethral Stricture Surgery (USS) PROM, facilitating a standardized outcome evaluation of voiding symptoms, health related QoL (HRQoL) and satisfaction with treatment [12]. A German language version of USS PROM with additional assessment of erectile function and urinary incontinence has recently been validated [13].

The aim of the present study was to evaluate in detail success, morbidity and HRQoL of BMGU for the treatment of urethral stricture disease in a contemporary, homogeneous patient cohort, using USS PROM.

## Methods

### Patient cohort

Data on 187 patients with urethral stricture disease, who were treated with BMGU at the University Medical Center Hamburg-Eppendorf between 12/05/2008 and 07/21/2010, were prospectively collected in the local urethroplasty database and retrospectively reviewed following Institutional Review Board approval (local ethics committee approval number PV4123). Patients were included following written consent from each patient. Inclusion criteria were patients with urethral stricture disease, who were treated with BMGU at our institution. Patients’ refusal to participate in the study was an exclusion criteria.

### Surgical procedure

BMGU has previously been described extensively [8, 13]. In brief, based on the location and length of the urethral stricture, single-stage or two-stage BMGU was performed [3, 5, 14, 15].

### Questionnaire

All patients received the validated German language version of the USS-PROM [13], addressing voiding symptoms, patients’ satisfaction and HRQoL including the following questionnaires: The International Consultation on Incontinence Questionnaire Male Lower Urinary Tract Symptoms (ICIQ-MLUTS) with an additional LUTS-specific QoL question from the ICIQ-MLUTSqol [16, 17]; The Peeling’s voiding picture; The EuroQuol 5D including pain evaluation and a visual analogue scale (VAS) [18]. In order to assess erectile function, the Erectile Function domain of the International Index of Erectile Function (IIEF-EF) [19] was added. On July 25th 2013, the questionnaire was sent to 187 patients in paper form by post. Patients received the questionnaire at home.

### Statistical analysis

The primary endpoint was the success rate following BMGU. Success was defined as stricture recurrence-free survival plus patients’ satisfaction with BMGU. Stricture recurrence was defined as any re-intervention and/or instrumentation following BMGU, including catheterization of the bladder or dilatation of the urethra. Patients’ satisfaction with BMGU was assessed with the question “Are you satisfied with the outcome of BMGU?” with the following answering possibilities: “very satisfied”, “satisfied”, “undecided”, “dissatisfied” and “very dissatisfied”. Only patients answering “very satisfied” or “satisfied” were classified as satisfied with BMGU. Erectile function, voiding symptoms, pain and health-related QoL were secondary endpoints of the present study. Stricture recurrence-free survival and success probabilities were assessed with the Kaplan-Meier method. Differences between groups were measured using the Logrank statistic. Univariable Cox regression was used to assess time to stricture recurrence. Associations between categorical variables were evaluated with the Fisher exact and χ^2^-tests. Differences in continuous variables were evaluated with the Mann-Whitney-U test (two categories) and the Kruskal-Wallis test (three or more categories). All tests are two-sided. A *p*-value of < 0.05 was defined to be statistically significant. All analyses were made with SPSS 23 (SPSS Inc., IBM Corp., Armonk, NY).

## Results

### Patients’ characteristics

In total, 187 patients underwent BMGU for urethral stricture disease between 12/05/2008 and 07/21/2010 at our center. Of these, 83 patients (44.4%) completed the questionnaire and were included in analyses. Table 1 presents clinical characteristics of the study cohort. In total, 69 patients (83.1%) had a stricture of the bulbar urethra. Single-stage BMGU was performed in 75 patients (90.4%). Prior surgical urethral interventions included direct visual internal urethrotomy, urethroplasty and dilatation in 59 patients (71.1%), 17 patients (20.5%) and 40 patients (48.2%), respectively.

**Table 1:** Clinical characteristics of 83 patients with urethral stricture disease treated with buccal mucosa graft urethroplasty

Age years [median (range)]	61 (16; 77)
Follow-up months [median (range)]	46 (36; 54)
Location of the stricture patients (%)	
Bulbar urethra	69 (83.1)
Penile urethra	13 (15.7)
Panurethral	1 (1.2)
Length of the stricture cm [median (range)]	5 (1; 16)
Procedure patients (%)	
Single-stage	75 (90.4)
Two-stage	8 (9.6)
Previous surgical urethral interventions patients (%)	63 (75.9)
Number of direct visual internal urethrotomies	
1	12 (14.5)
2–5	38 (45.8)
> 5	9 (10.8)
Number of urethroplasties	
1	14 (16.9)
> 1	3 (3.6)
Number of dilatations	
≥ 1	40 (48.2)

### Outcome

**Fig. 1:**
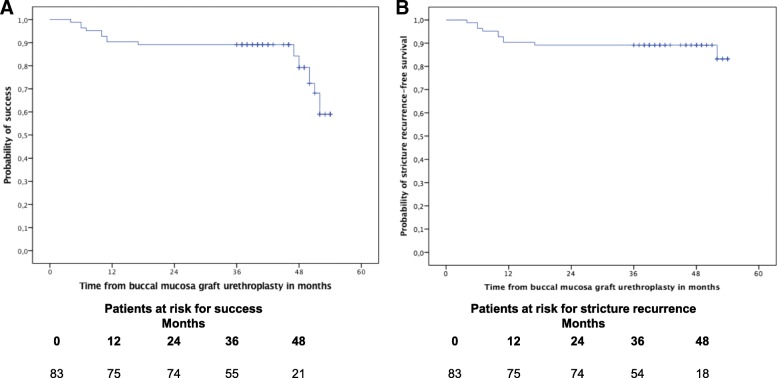
Kaplan-Meier plots of success (**a**) and stricture recurrence-free survival (**b**) in 83 patients with urethral stricture disease treated with buccal mucosa graft urethroplasty

**Fig. 2:**
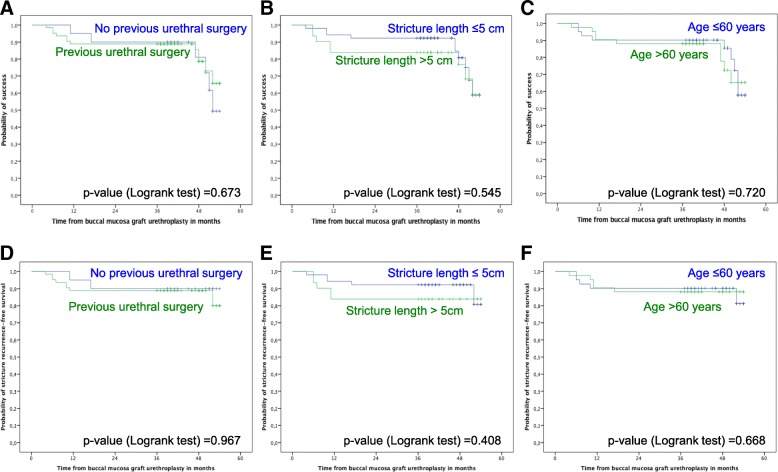
Kaplan-Meier plots of success (**a**, **b**, **c**) and stricture recurrence-free survival (**d**, **e**, **f**) in 83 patients with urethral stricture disease treated with buccal mucosa graft urethroplasty stratified by previous urethral surgery status (**a**, **d**), stricture length (**b**, **e**) and age (**c**, **f**)

At a median follow-up of 46 months (range: 36–54 months), success was found in 65 patients (78.3%), and 73 patients (88.0%) had no stricture recurrence (Fig. 1). There was no difference in the probability of success and stricture recurrence-free survival among patients with and without previous surgical urethral interventions, according the stricture length stratified by ≤5 cm and > 5 cm, as well as according patients’ age stratified by ≤60 years and > 60 years (Fig. 2).

Following BMGU, post-micturition dribbling and streaking the urethra was observed in 45 patients (54.2%) and 39 patients (46.1%), respectively. Severe penile shortening, penile curvature and hypoesthesia of the glans or scrotum was reported by 2 patients (2.4%), respectively. Fifty patients (60.3%) had erections with normal or slightly reduced rigidity. Forty- three patients (51.8%) had a normal or slightly reduced ejaculate volume and 9 patients (10.8%) had painful sensations during ejaculation. In total, 7 patients (8.4%) described a strong impairment of sexuality.

Table 2 displays data on erectile function, voiding symptoms, pain and QoL. Based on USS PROM, 67 patients (80.7%) and 68 patients (81.9%) reported improvement of HRQoL and satisfaction with the surgical procedure, respectively. There was no difference in ICIQ-MLUTS, Peeling’s voiding picture, IIEF-EF, ICIQ-MLUTSqol and EQ-5D among patients with and without previous surgical urethral interventions as well as according the stricture length stratified by ≤5 cm and > 5 cm (data not shown). There was no difference in ICIQ-MLUTS, Peeling’s voiding picture, ICIQ-MLUTSqol and EQ-5D among patients with an age ≤ 60 years and > 60 years (data not shown). Patients ≤60 years of age had elevated IIEF-EF scores compared to patients > 60 years of age (median IIEF-EF in patients ≤60 years vs. > 60 years: 29 vs. 3; *p*-value≤0.001).

**Table 2:** Erectile function, voiding symptoms, pain and health related quality of life of 83 patients with urethral stricture disease treated with buccal mucosa graft urethroplasty

Voiding symptoms	
ICIQ-MLUTS score [median (range)]	6 (0; 17)
Peeling’s voiding picture [median (range)]	2 (1; 4)
Erectile function	
IIEF-EF score [median (range)]	22 (1; 30)
Health related quality of life	
ICIQ-MLUTSqol	
Urinary symptoms interfere with life – a little or not at all [patients (%)]	68 (81.9)
EQ-5D: severe pain postoperatively [patients (%)]	1 (1.2)
EQ-5D VAS [median (range)]	80 (20; 100)
Surgical outcome	
USS PROM: improvement of quality of life [patients (%)]	67 (80.7)
USS PROM: satisfaction with surgical procedure [patients (%)]	68 (81.9)

### Risk factor analysis for reduced success and stricture recurrence

In univariable Cox regression analysis, neither previous surgical urethral interventions, nor stricture length, nor patients’ age were associated with a reduced success or increased stricture recurrence following BMGU (data not shown).

## Discussion

We found that BMGU offers excellent outcome in a contemporary patient cohort with urethral stricture disease. Nearly 90% of patients did not require any form of re-intervention or instrumentation at a maximum follow-up exceeding 4 years, which is in line to results of earlier studies with 66 to 100% stricture recurrence-free survival at 15 to 83 months follow-up [7]. Based on strict success criteria, we found that the success rate of 78% was lower than the stricture recurrence-free survival. A reason for this discrepancy may certainly be the fact that outcome measure comprising the factor patients’ satisfaction may facilitate a more patient-oriented evaluation of the success of BMGU. Thus, our findings emphasize the importance of the inclusion of PROM in the evaluation of BMGU outcome. Indeed, although PROM does not allow an objective assessment of surgical complications, it adds crucial information on patients’ subjective morbidity, including voiding symptoms, erectile function, pain and health-related QoL following BMGU.

We found favorable Peeling’s voiding picture and ICIQ-MLUTS scores following BMGU, which corresponds to earlier reports [8, 13, 20]. Conversely, voiding symptoms including post-micturition dribbling and streaking the urethra were quite frequently reported in the present study. This discrepancy may indicate that patients do not consider these symptoms to be bothering. Indeed, based on ICIQ-MLUTSqol, more than 80% of patients reported that urinary symptoms did not interfere with life, which is comparable to the results of earlier studies [20]. The median IIEF-EF score showed mild erectile dysfunction, and a relevant proportion of patients reported erections with reduced rigidity. Other impairments of sexual function comprised reduced ejaculate volume, painful ejaculation, penile shortening and curvature as well as hypoesthesia of the glans or scrotum, which corresponds to previously reported findings [8, 21, 22]. However, more than 90% of patients did not report a strong deterioration of their sexuality. Other authors have previously shown variable impairment of sexual function and IIEF scores following BMGU [8, 13, 23–25]. Heterogeneity across different studies may be due to variable evaluation methods of sexual and erectile function, including inconsistent implementation of IIEF [21, 22, 26]. Importantly, our findings emphasize again the need of a standardized assessment of sexual and erectile function following BMGU. We found that the majority of patients did not experience strong pain postoperatively, which is reflected by low EQ-5D VAS scores and corresponds to earlier findings [8, 13, 20]. In accordance to a favorable postoperative morbidity, more than 80% of patients described an improvement of HRQoL and satisfaction with BMGU.

We did not find differences in success and morbidity among patients with and without previous urethral interventions as well as according to stricture length and patients’ age. Not surprisingly, these factors were not associated with an increased risk for failure and stricture recurrence in univariable analysis. In contrast, other authors have previously identified prior urethral interventions, stricture length and age as potential independent predictors for stricture recurrence and failure [27–29]. Variable findings across studies may be due to differences in cohort sizes, surgical urethroplasty techniques, follow-up and the definition of success. For example, patients with posterior urethral stricture were excluded from analyses [28] and stricture recurrence-free survival was assessed by urethrocystoscopy and symptom inquiry [29]. In fact, further studies are needed to clearly define risk factors for stricture recurrence and failure following BMGU.

The present study has relevant limitations. First and foremost are limitations inherent to the retrospective study design including a limited follow-up. Patients did not receive USS PROM preoperatively. Thus, baseline data on ICIQ-MLUTS, Peeling’s voiding picture, IIEF-EF, ICIQ-MLUTSqol, EQ-5D and VAS was not available. This represents an important limitation of the present study, since a comparison of preoperative and postoperative voiding, erectile function, urinary continence and quality of life was not possible. Thus, there remains a risk of bias regarding the impact of urethroplasty on patient reported outcomes. In addition, the response rate is limited and to draw conclusion regarding the patients who did not answer the questionnaire is not possible, which might represent a source of bias. The specific reasons for the limited response rate remain unknown. Patients received the questionnaire at variable time points following BMGU, which might have influenced the results, since the length of follow-up seems to be a predictor for stricture recurrence [27]. Data on buccal mucosa donor site complications were not available, although donor site morbidity may certainly have a detrimental impact on patients’ satisfaction and QoL [30]. Moreover, the present study did not comprise instrument-based outcome measure, i,e. urethral calibration, uroflowmetry, urethrography and urethrocystoscopy. These techniques may allow a more surgery-oriented outcome evaluation. In addition, instrument-based outcome measure and comparison to baseline values allows objective evaluation of surgical results. Missing data on urethral calibration, uroflowmetry, urethrography and urethrocystocopy following BMGU represents a limitation of the present study. Urethral calibration can identify narrowing of the urethral diameter and may detect stricture recurrence early following urethroplasty. However, stricture recurrences have to narrow the urethra to a caliber <10F to result in a relevant decrease of urinary flow rates [7]. In addition, urethrography implicates the use of radiation, and urethrocystoscopy is an invasive procedure, which may be problematic for monitoring patients with USD. Finally, urethral calibration, uroflowmetry, urethrography and urethrocystoscopy may not allow assessing adequately the morbidity of BMGU, which was a secondary endpoint of the present study. The present study did focus on success, morbidity and HRQoL of urethroplasty. Therefore, the utilization of USS-PROM is appropriate, since it represents a validated instrument allowing standardized patient-orientated outcome evaluation of success, voiding symptoms, quality of life and satisfaction.

## Conclusions

In patients with urethral stricture disease, BMGU offers excellent outcome, success, morbidity and QoL, independently of previous urethral interventions, patients’ age and stricture length. Prior surgery, patients should be counseled on morbidity of BMGU, including voiding symptoms, pain and impairment of sexual and erectile function. USS PROM allows a detailed and standardized analysis of success, morbidity and HRQoL, and should therefore be consistently utilized in outcome reporting of BMGU.

## Abbreviations

EQ-5D VAS: Euro QoL 5D Visual Analogue Scale
EQ-5D: Euro QoL 5D
ICIQ-MLUTS: Incontinence Questionnaire Male Lower Urinary Tract Symptoms Module
ICIQ-MLUTSqol: LUTS-specific QoL Question of Incontinence Questionnaire Male Lower Urinary Tract Symptoms Module
IIEF-EF: Erectile Function domain of the International Index of Erectile Function
PROM: Patient Reported Outcome Measure
QoL: Quality of life
